# Analysis of pathogenic bacteria and antimicrobial residues in bovine waste milk on dairy farms in southern Chile

**DOI:** 10.3389/fvets.2025.1613185

**Published:** 2025-08-01

**Authors:** Fernando Ulloa, Martina Penati, José M. Hernández-Agudelo, Carlos Tejeda, Pamela Steuer, María Filippa Addis, Armin Mella, Nivia Canales, Juan Pablo Soto, Miguel Salgado

**Affiliations:** ^1^Facultad de Ciencias Veterinarias, Instituto de Medicina Preventiva Veterinaria, Universidad Austral de Chile, Valdivia, Chile; ^2^Escuela de Graduados, Facultad de Ciencias Veterinarias, Universidad Austral de Chile, Valdivia, Chile; ^3^Department of Veterinary Medicine and Animal Sciences, University of Milan, Lodi, Italy; ^4^Facultad de Ciencias, Instituto de Bioquímica y Microbiología, Universidad Austral de Chile, Valdivia, Chile; ^5^Prolesur Los Lagos, Los Lagos, Chile

**Keywords:** antimicrobial resistance, calves, calf disease, grazing, ESBL

## Abstract

Waste milk (WM), a byproduct of dairy production, is often used as a cost-effective feed for calves, but it can contain pathogens and antimicrobial residues, which pose health risks. This study examined the microbiological quality and the presence of antimicrobial residues in WM from 36 dairy farms in southern Chile. In a cross-sectional study, WM samples were collected, and farm management data were gathered through a questionnaire. The samples were analyzed for total bacterial load, coliforms, staphylococci, streptococci/streptococci-like organisms (SSLOs), *Salmonella* spp., *Mycobacterium avium* subsp. *paratuberculosis* (MAP), *Mycobacterium bovis*, *Mycoplasma* spp., *Prototheca* spp., methicillin-resistant *Staphylococcus aureus* (MRSA), and extended-spectrum beta-lactamase (ESBL)-producing *E. coli*. Antimicrobial residues were detected using a commercial test. A high average bacterial load (7.63 × 10^6^ CFU/mL) and significant levels of staphylococci, SSLOs, and coliform were found. In addition, *S. aureus* (33.3%), MAP (11.1%), and *Salmonella* spp. (2.8%) were detected. ESBL-*E. coli* was found on five farms, with *bla*_CTX-M_ being the predominant gene. Antimicrobial residues, mainly beta-lactams, were present in 55.6% of samples. These results show that WM in this region frequently contains pathogens, antimicrobial residues, and resistant bacteria. Current farm practices, such as feeding untreated WM to calves, may contribute to the spread of antimicrobial resistance and compromise calf health.

## 1 Introduction

Optimal nutrition for calves in early life is essential for growth, the development of the immune system, and future performance. While commercially available milk replacers and/or saleable milk represent the ideal liquid feed for pre-weaned calves, economic pressures often lead dairy producers to use waste milk (WM) (1). Waste milk encompasses milk from cows with intramammary infections (IMIs) treated with antibiotics, milk contaminated with other drugs, milk from cows with clinical mastitis (i.e., that which contains pus, fibrin, or effusions), milk with high somatic cell counts (SCCs), and post-colostral transition milk (2). The prohibition of its sale for human consumption is mandatory, and this represents a significant economic loss (3).

Despite reported concerns, WM is widely used as a calf feed due to its nutritive value and cost-effectiveness compared to milk replacers (1, 4). However, feeding raw WM to calves could be problematic due to the potential presence of pathogens and antibiotic residues (5, 6), which potentially increases the risk of disease and antimicrobial resistance (2, 7). Furthermore, WM has been identified as a vehicle for pathogens that pose a threat to both animal and public health, such as *Mycobacterium avium* subsp. *paratuberculosis* (MAP) (8), *Mycobacterium bovis* (9), *Salmonella* spp. (10), and *Mycoplasma* spp. (11, 12). The most comprehensive studies on bacterial contamination in WM identified *Streptococcus* spp. and members of the *Enterobacteriaceae* family as the predominant bacterial groups (5, 6).

Beyond its general composition, WM frequently harbors mastitis-causing pathogens. *Staphylococcus aureus*, for instance, is a primary agent of contagious bovine mastitis globally (13, 14), known for variable cure rates and its association with antimicrobial resistance (AMR) (15). The presence of methicillin-resistant *S. aureus* (MRSA) in dairy environments is a significant One Health concern due to its role in multidrug-resistant human infections (16). Furthermore, the dissemination of AMR through WM is a critical issue, with particular concern surrounding bacteria producing extended-spectrum beta-lactamases (ESBLs). These enzymes confer resistance to beta-lactam antibiotics, including penicillins and third- and fourth-generation cephalosporins, thereby complicating treatment options in both human and veterinary medicine, and their detection in dairy cattle is increasingly reported (17).

Studies have shown mixed results regarding the effects of WM on calf health and performance. While some research reported no significant differences in growth, intake, or health parameters between calves fed WM and those fed pasteurized WM or bulk milk (18), others reported a higher incidence of diarrhea and changes in fecal microbiota in calves fed with WM containing antibiotic residues (19, 20). The use of WM, particularly unpasteurized WM containing antibiotic residues, remains controversial due to its potential long-term impact on calf health, antimicrobial resistance (7, 19), and environmental dissemination via manure, soil contamination, and the release of antimicrobials in bovine slurry (21).

At the local level, it has been estimated that 51.7% of the dairies in the Los Ríos Region (Southern Chile) use untreated WM to feed calves (22). This region’s reliance on predominantly grazing-based dairy systems (23), which differ significantly from the confinement systems where most previous comprehensive WM studies were conducted (5, 6), may pose unique challenges and contamination profiles. Therefore, it is important to assess the quality of WM in this specific context to understand the risks to the calf and public health. Hence, this study aimed to analyze the presence of pathogenic bacteria and antimicrobial residues in WM samples collected from dairy farms in southern Chile.

## 2 Materials and methods

### 2.1 Study population and sampling of waste milk

A cross-sectional study was conducted between July and November 2023 to investigate the composition of pathogenic bacteria and antimicrobial residues in bovine WM in the Los Ríos and Los Lagos regions in southern Chile. A convenience sampling method was used to select 36 dairy herds from a list provided by 2 dairy manufacturing companies. In total, 36 WM samples were collected from storage containers either at room temperature or under refrigeration, before and after treatment (pasteurization or acidification), according to the specific management practices of each farm. To ensure sample representativeness, each sample was homogenized either using an automated stirrer for 10 min or manually with a sterile steel spoon. Subsequently, 500 mL of WM was transferred to sterile glass bottles and immediately transported to the laboratory under refrigerated conditions (0–4°C). Ethical approval was obtained from the Universidad Austral de Chile Bioethics Committee (Protocol No. 516–2023).

### 2.2 Farm description

A questionnaire was administered to each participating farm on the day of sampling to collect data on farm management practices. The questionnaire consisted of 23 questions, covering general farm information, calf housing, feeding practices, health management protocols (including antimicrobial use), and biosecurity measures (Supplementary material).

### 2.3 Microbiological analysis

This study involved the detection and quantification of bacterial populations relevant to milk quality and animal health. The general microbiological quality of WM was assessed by quantifying total bacteria, total coliforms, staphylococci, streptococci, and streptococci-like organisms (SSLOs), a group that includes members of the genera *Streptococcus*, *Enterococcus*, and *Lactococcus*, among others. For this purpose, 100 μL of 10-fold serial dilutions of each milk sample were plated in duplicate on different culture media: plate count agar (Oxoid, Hampshire, United Kingdom) for total bacterial count (TBC), MacConkey agar (Oxoid, Hampshire, United Kingdom) for total coliforms, mannitol salt agar (Oxoid, Hampshire, UK) for staphylococci, and Edwards medium (Oxoid, Hampshire, United Kingdom) supplemented with 5% sheep blood for SSLOs. All plates were incubated at 37°C for 24 h. Bacterial concentrations were determined by counting the typical colonies for each bacterial group and multiplying by the corresponding dilution factor.

In addition, specific pathogens, including *Salmonella*, *Prototheca*, and *Mycoplasma* spp., were tested for following the protocol described by Ulloa et al. (24). For *Prototheca* spp., 100 μL of each milk sample was inoculated into 5 mL of Prototheca isolation medium (PIM) broth and incubated at 37°C for 24 h. After incubation, a 100-μL of aliquot of the broth was plated onto PIM agar and incubated at 37°C for 72 h. For the detection of *Salmonella* spp., 100 μL of each milk sample was seeded into 5 mL of selenite cystine broth (Oxoid, Hampshire, United Kingdom) and incubated at 37°C for 16 h. A 100 μL of aliquot of the enriched broth was then plated onto xylose lysine deoxycholate (XLD) agar (Oxoid, Hampshire, UK) and incubated at 37°C for 48 h. Suspected isolates were confirmed using a qPCR protocol (25). *Mycoplasma* spp. detection was performed by inoculating 100 μL of each milk sample onto a modified Hayflick Medium (Oxoid, Hampshire, United Kingdom), followed by incubation at 37°C for 12 days in an atmosphere containing 10% CO₂.

To detect viable MAP and *Mycobacterium bovis*, a DNA extraction procedure based on phage-mediated separation was performed (26). MAP and *M. bovis* were confirmed by qPCR, targeting the IS*900* sequence and the *RD4* gene, respectively (26, 27).

### 2.4 MRSA and ESBL-*E. coli* detection

The presence of two antimicrobial-resistant bacteria, extended-spectrum beta-lactamase (ESBL)-producing *Escherichia coli* (ESBL-*E. coli*) and methicillin-resistant *Staphylococcus aureus* (MRSA), was assessed following the protocol described by Penati et al. (17) and Fusar-Poli et al. (16). In short, to enhance bacterial recovery, an enrichment step was performed using Müller–Hinton broth for ESBL-*E. coli* and Müller–Hinton broth supplemented with 6.5% NaCl (Oxoid, Hampshire, United Kingdom) for MRSA. Samples were incubated at 37°C for 24 h. Following enrichment, a 50-μL aliquot of the broth was plated onto CHROMagar™ ESBL or CHROMagar™ MRSA selective media (CHROMagar, Paris, France) and incubated under the same conditions. From each plate, three suspected colonies were selected for species identification by PCR, following the protocols described by Frahm and Obst (28) for *E. coli* and by Baron et al. (29) for *S. aureus*. ESBL-*E. coli* confirmation was performed using the double-disk synergy test (DDST) (30) and PCR detection of *bla* genes associated with ESBL expression (*bla*_SHV_, *bla*_TEM_, and *bla*_CTX-M_) (31). MRSA confirmation was performed using a PCR assay targeting the *mecA* gene (32).

### 2.5 Detection of antimicrobial residues

All milk samples were tested for antimicrobial residues using the IDEXX SNAPduo™ ST Plus Test rapid test (Idexx Laboratories Inc., Westbrook, ME, United States). This test detects beta-lactam antimicrobials, cephalosporins, and tetracyclines by binding them to enzyme-linked receptors. The reading was carried out according to the manual. The sensitivity of the test used in the penicillin group is 2–4 ppb. For the cephalosporin group, it is 8–60 ppb, and for the tetracycline group, it is 16–40 ppb.

### 2.6 Data analysis

Statistical analyses were performed using R software version 3.1.2 (57). Descriptive statistics (means, standard deviations, medians, interquartile ranges, and proportions) were calculated to summarize farm characteristics and WM composition.

## 3 Results

### 3.1 Farm characteristics and management

Regarding general farm characteristics, the average number of milking cows per farm at the time of sampling was 394 (range 14–1,880). Most farms (83.3%) used a grazing-based production system with predominantly bi-seasonal (47.2%) or strictly seasonal (30.6%) calving, concentrated in autumn and spring or only in spring, respectively. Based on annual milk production, farms were categorized as small (<500,000 kg), medium (500,000–1,500,000 kg), and large (>1,500,000 kg). In the study sample, these categories represented 28, 28, and 44% of the farms, respectively. The bulk tank SCC average, obtained from the farm’s weekly records, was 279 × 10^3^ cells/mL (range 61–1,540 × 10^3^ cells/mL).

### 3.2 Calf management

At the time of sampling, the farms had an average of 114 calves (range 4–700), with an average of 17 calves on small farms, 45 on medium-sized farms, and 218 on large farms. Calves were housed in collective pens on 77.8% of the farms, while 22.2% used a mixed system, in which newborns were initially placed in individual pens for 5–15 days, before being transferred to collective pens.

In terms of colostrum management, 72.2% of the farms provided colostrum directly from the dam, while 22.2% used colostrum banks. Most farms (80.5%) did not treat WM before feeding it to calves, while 16.7% used acidification, and one farm (2.8%) carried out pasteurization. Additionally, 72% of the farms took no account of calf age when taking the decision to use WM for feeding, and 75.0% took no account of sex. The most common feeding method was via individual or collective buckets (91.7%), with only 8.3% (three farms) using automatic feeders. The majority (94%) cleaned the equipment after each use, while a small proportion cleaned it once a day (2.8%) or less frequently (2.8%).

Regarding health management, 66.7% of the farms separated sick calves from healthy ones. In 44.4% of farms, disease diagnoses were established by the calf caretaker. In 25% of farms, this was done by the farm manager, in another 25%, most commonly in large farms, this was done by the veterinarian, and in 5.6%, this was done by others.

Regarding antimicrobial administration to calves, this was primarily handled by the calf caretaker (55.6% of the farms), followed by the farm manager (25%), the veterinarian (11.1%), and by others (8.3%). The involvement of veterinarians in antimicrobial administration was observed mainly on large farms with permanent veterinary staff (three farms) and on one small farm owned by a veterinarian.

### 3.3 Antimicrobial management

Most of the farms surveyed kept records of both antimicrobial purchases (80.6%) and calf treatments (80.6%). However, while most farms had protocols for antimicrobial use in calves, a considerable proportion (41.7%) lacked formal guidelines. For respiratory infections in calves, the most commonly used antimicrobials, either alone or in combination, were tetracyclines (53%), followed by fluoroquinolones (39%), and florfenicol (22%). For diarrhea, sulfonamides were the most frequently used (69%), followed by fluoroquinolones (39%) and tetracyclines (22%).

Regarding antimicrobial use in adult cattle, cephalosporins were the most commonly used class for treating clinical mastitis (64% of farms), with 27.8% of all farms using first-generation, 47.2% using third-generation, and 27.8% using fourth-generation cephalosporins. Some farms used more than one generation. Non-cephalosporin beta-lactams (e.g., penicillins) were the second most used class (44% of farms), followed by tetracyclines (19%). For dry cow therapy, 39% of farms used a combination of a beta-lactam and an aminoglycoside, while 28% used cephalosporins and 28% used a non-cephalosporin beta-lactam.

### 3.4 Microbiological analysis

The average TBC in WM samples was 7.63 × 10^6^ CFU/mL (ranging from 1.0 × 10^2^ to 1.42 × 10^8^ CFU/mL). Notably, seven samples had TBC levels below 1.0 × 10^4^ CFU/mL, while 16 had counts below 1.0 × 10^5^ CFU/mL. The average staphylococcal count was 2.07 × 10^4^ CFU/mL (ranging from 0 to 6.6 × 10^5^ CFU/mL), with three samples showing no detectable staphylococci. The average SSLO count was 3.90 × 10^5^ CFU/mL (ranging from 0 to 8.9 × 10^6^ CFU/mL). The total coliform count average was 2.18 × 10^4^ CFU/mL (ranging from 0 to 2.9 × 10^5^ CFU/mL).

In terms of specific pathogens, *S. aureus* was detected in samples from 12 farms (33.3%)*, Salmonella* spp. was detected in samples from one farm (Farm 17), viable MAP was identified in samples from four farms (1, 14, 16, and 33), and *Mycoplasma* spp., *Prototheca* spp., and *Mycobacterium bovis* were not found in any samples.

### 3.5 Detection of MRSA and ESBL-*E. coli*

No MRSA was detected in any of the WM samples; however, 13 isolates of ESBL-*E. coli* were identified in 5 of the 36 farms (farms 7, 16, 18, 20, and 33). A molecular analysis revealed the presence of *bla*_CTX-M_ in 11 out 13 isolates, while *bla*_TEM_ was detected in 3 isolates. Two isolates from Farm 18 harbored both *bla*_CTX-M_ and *bla*_TEM_. The *bla*_SHV_ gene was not detected in any of the isolates. The specific ESBL gene profiles for each isolate are summarized in Table 1.

**Table 1:** Distribution of ESBL genes in *E. coli* isolates from waste milk samples.

Farm	Number of isolates	*bla*_CTX-M_	*bla*_TEM_	*bla*_SHV_
Farm 7	1	−	−	−
Farm 16	3	+	−	−
Farm 18	2	+	+	−
	1	−	+	−
Farm 20	3	+	−	−
Farm 33	3	+	−	−

### 3.6 Antimicrobial detection by SNAP test

The SNAP test detected antimicrobial residues in 22 out of 36 (61.1%) of the WM samples (Table 2). Beta-lactams alone were the most frequently detected residues, present in 50.0% of the samples. A combination of beta-lactam and tetracycline was found in two (5.5%) of the samples, while one (2.8%) contained a combination of beta-lactam, tetracycline, and cephalexin. Tetracycline was detected alone in one (2.8%) of the samples. Thirteen of the samples (36.1%) tested negative for antimicrobial residues. One sample (2.8%) yielded an invalid result due to excessive density, which prevented it from properly passing through the SNAP test membrane.

**Table 2:** Detection of antimicrobial residues in waste milk samples using the SNAP test.

Antimicrobial	Number of farms (%)
Beta-lactam*	18 (50.0)
Negative	13 (36.1)
Beta-lactam + Tetracycline	2 (5.5)
Beta-lactam + Tetracycline + Cephalexin	1 (2.8)
Tetracycline	1 (2.8)
Invalid	1 (2.8)
Total	36 (100.0)

*Beta-lactams include penicillin, amoxicillin, and cloxacillin, among others.

## 4 Discussion

This study was conducted on dairy farms in southern Chile, a region where seasonal or bi-seasonal calving systems are common; these systems concentrate calving into a period of high labor demand and potential hygiene challenges (33). Data and sample collection coincided with this peak calving season (July–September), a time when the volume of WM is at its highest, calving pens are intensively used, and the physiological stress on cows is high (34). These factors, particularly in group maternity areas, create an environment conducive to increased bacterial shedding and pathogen transmission, which may significantly increase the risks associated with feeding untreated WM to calves.

Several common management practices observed on the surveyed farms further exacerbated these risks. Of particular concern was the fact that 80.6% of farms used untreated WM for feeding, thereby directly exposing susceptible calves to high levels of bacteria, pathogens, and antimicrobial residues. Collective housing, while potentially offering welfare benefits (35), was also widespread. When combined with the failure to isolate sick calves—observed on 33% of farms—this practice can facilitate pathogen spread (35, 36). Additionally, the practice of feeding colostrum directly from the dam without testing and the lack of age and sex differentiation in WM feeding increase the risk of both vertical and horizontal transmission of pathogens such as *S. aureus* and MAP (9, 37).

Practices of antimicrobial usage on the surveyed farms pose serious risks of resistance development and suggest the need for improved stewardship. While 80.6% of farms recorded antimicrobial use and veterinarians were often consulted, the lack of formal, written protocols on 41.7% of farms, coupled with reliance on calf caretakers for diagnosis and treatment, may increase the likelihood of inappropriate antimicrobial use. Therefore, targeted training of farm personnel on the principles of prudent antimicrobial use is necessary. The frequent use of fluoroquinolones in calves and third- and fourth-generation cephalosporins for mastitis in cows, both of which are critically important antimicrobial classes (38), directly contributes to antimicrobial residues in WM. This exposes calves to subtherapeutic drug levels, promoting the selection and spread of resistant bacteria, including ESBL-*E. coli* (39, 40).

The microbiological analysis indicated that the hygienic quality of WM, while highly variable, was generally poor. These high bacterial loads pose a considerable risk to calf health. The TBC (7.63 × 10^6^ CFU/mL) and total coliforms (2.18 × 10^4^ CFU/mL) were significantly higher than those reported in previous studies (5, 6). These elevated counts indicate inadequate hygiene practices during milk collection, storage, or handling and increase the risk of both reduced nutritional value and enteric infections in calves (1, 22). *Staphylococcus* spp. and SSLOs, common mastitis-associated organisms, were also present at considerable levels, which was consistent with previous findings in studies on WM (5, 6).

*Salmonella* spp. was identified in one sample. Although the frequency was low, the detection of *Salmonella* spp. is significant due to its zoonotic potential and capacity to cause severe gastrointestinal illness in humans (10). Previous studies have reported mixed findings regarding the presence of *Salmonella* in WM; Selim and Cullor (5) did not detect it, while Edrington et al. (41) successfully isolated it from WM. However, this last study concluded that milk-borne *Salmonella* is not a major transmission route for neonatal calves and that pasteurization does not significantly influence the fecal shedding of this pathogen.

The detection of viable MAP in four WM samples was particularly concerning, as MAP is the causative agent of Johne’s disease, a chronic granulomatous enteritis in ruminants that results in substantial economic losses (42). The presence of viable MAP in WM indicates a high risk of transmission to susceptible calves and perpetuation of the disease within the herd. This is particularly significant in Chile, where herd-level MAP prevalence has been reported between 44 and 87% (43). Significantly, two of the MAP-positive WM samples originated from farms that used acidification as a treatment method. While acidification can reduce bacterial loads (12), MAP is highly resistant to low pH and other environmental stressors (44). This resilience is consistent with previous findings showing that pasteurization only partially inactivates MAP (45). These results demonstrate the limitations of acidification as a sole control strategy for MAP transmission in WM. Consequently, pasteurization remains the recommended approach for reducing the risk of MAP transmission, despite not guaranteeing complete inactivation (10). Acidification remains an economical alternative for smaller dairy operations, as it can reduce bacterial loads when pH remains within the effective range. Studies indicate that acidification can significantly reduce TBC, *Salmonella*, and *Mycoplasma*, but its effectiveness depends on incubation time and pH control (12). Therefore, strict process monitoring is essential to maximize efficacy. The presence of *Salmonella* in one sample of acidified WM may indicate that the process had not been controlled properly.

Neither *Mycobacterium bovis* nor *Mycoplasma* spp. were detected in the WM samples. The absence of *Mycobacterium bovis* is consistent with southern Chile’s low herd-level prevalence (0.3%) and within-herd prevalence (0.67%), which is attributed to a long-standing national control program (46).

The detection of antimicrobial residues in 61.1% of WM samples, with a predominance of beta-lactams (50.0%), suggests a significant risk associated with this feeding practice. Similar, and even higher, prevalences of antimicrobial residues have been reported in other studies, with 63% testing positive with ELISA (5), 82.3% using the SNAP test (47), and 60% using LC–MS/MS (6). The presence of such residues, even at low levels, can disrupt the calf gut microbiome (19) and provide a selective environment that favors the growth of resistant bacteria, including ESBL-producing *E. coli* (48, 49). ESBLs are *β*-lactamases that can hydrolyze expanded-spectrum cephalosporins (such as cefotaxime, ceftriaxone, ceftazidime, or cefepime) and monobactams, which are important antimicrobials for animals and humans (50). Studies have reported high prevalence rates of ESBL/AmpC-*E. coli* in pre-weaned calves (63.5%) (51) and bulk tank milk (9.5%) (52) on German dairy farms. The presence of antibiotic residues, especially cefquinome, in WM is associated with an increased occurrence of ESBL-producing bacteria (39, 49). Feeding WM to calves has been identified as a significant risk factor for ESBL-*E. coli* colonization in calves (51, 53). The role of the presence of ESBL-*E. coli* in WM in its colonization of the calf gut is not clear, but direct transmission of viable ESBL-*E. coli* to calves through contaminated colostrum has recently been demonstrated (54).

ESBL enzymes are diverse, with TEM, SHV, and CTX-M representing the major families. The predominance of the *bla*_CTX-M_ gene in our isolates is consistent with global epidemiological patterns, where *bla*_CTX-M_ has become the most common ESBL type (50). While we did not identify specific *bla*_CTX-M_ variants, common types found in livestock include *bla*_CTX-M-1_, *bla*_CTX-M-2,_ *bla*_CTX-M-9_, *bla*_CTX-M-14_, *bla*_CTX-M-15_, *bla*_CTX-M-32_, and *bla*_CTX-M-55_, of which *bla*_CTX-M-14_ and *bla*_CTX-M-15_ are also frequently found in human infections (55). Two isolates in our study contained both *bla*_CTX-M_ and *bla*_TEM_, potentially conferring an even broader resistance spectrum. Notably, one isolate from Farm 7, phenotypically confirmed as ESBL-producing, did not yield a positive result for any of the targeted *bla* genes. This is not uncommon, as some phenotypically confirmed ESBL-producing isolates may harbor other less common ESBL gene variants not covered by the primers used, or in rare cases, may represent false positives in phenotypic testing (56). The frequent use of third- and fourth-generation cephalosporins on these farms, which is documented in our survey, is a known driver of ESBL selection (39, 40, 49). Further research, including *bla*_CTX-M_ variant identification and longitudinal studies, is crucial to understanding local ESBL epidemiology and transmission dynamics in calves.

The cross-sectional design of this study captures a single point in time and cannot establish causal relationships between farm practices and observed outcomes. While the sampled farms represent a substantial portion of regional milk production, the convenience sampling method may not provide us with results that are generalizable to all Chilean dairy operations. Nevertheless, the findings show the risks associated with current WM management in southern Chile. Widespread bacterial contamination, the presence of potentially zoonotic pathogens (including *Salmonella* and MAP), and the high frequency of antimicrobial residues and ESBL-producing *E. coli* pose a significant threat to calf health and potentially to public health.

## 5 Conclusion

This study found a high frequency of antimicrobial residues and significant bacterial contamination, including pathogenic species and ESBL-producing *E. coli*, in WM samples from dairy farms in southern Chile. While the general risks of feeding WM are known, this research offers a novel contribution by providing the first comprehensive characterization of WM contaminants within the predominantly grazing-based dairy systems. These findings show the potential risks associated with the common practice of feeding untreated WM to calves, particularly regarding the development of antimicrobial resistance and the potential for zoonotic disease transmission. Consequently, it is recommended to implement WM pasteurization to reduce pathogen load, alongside comprehensive antimicrobial stewardship programs designed to address both residue and resistance issues.

## Data Availability

The raw data supporting the conclusions of this article will be made available by the authors, without undue reservation.
