# Supplementary material for: Analysis of pathogenic bacteria and antimicrobial residues in bovine waste milk on dairy farms in southern Chile
Source: Front Vet Sci. 2025 Aug 1;12:1613185. doi: 10.3389/fvets.2025.1613185 (PMC12355818; doi:10.3389/fvets.2025.1613185)
Supplement: Supplementary file 1 [file Table_1.docx]

Supplementary Material

**Supplementary Table 1.** General characteristics of the farms

| **Question** | **Answer** |
| --- | --- |
| Cows in milk  0-49  50-99  100-299  300-1,000  >1,000 | 394 (Range 14-1,880)  8 (22.2%)  2 (5.6%)  10 (27.8%)  14 (38.9%)  2 (5.6%) |
| Calving system  Seasonal  Continuous  Bi-seasonal | 11 (30.6%)  8 (22.2%)  17 (47.2%) |
| Production system  Grazing  Confined  Mixed | 30 (83.3%%)  1 (2.8%)  5 (13.9%) |
| Annual milk production (kg)  <500,000  500,000-1,500,000  >1,500,000 | 10 (27.8%)  10 (27.8%)  16 (44.4%) |
| Bulk tank SCC average (cells/mL ×10^3^) | 279 (61-1,540) |

**Supplementary Table 2.** Characterization of calves management

| **Question** | **Answer** |
| --- | --- |
| Numbers of calves at the visit | 114 (Range 4-700) |
| Type of calf housing (individual pens, group pens, etc.):  Individual  Collective  Mixed | 0 (0%)  28 (77.8%)  8 (22.2%) |
| What is the source of colostrum?  Dam  Colostrum bank  Mixed | 26 (72.2%)  8 (22.2%)  2 (5.6%) |
| If you feed calves WM, what treatment do you use?  Pasteurization  Acidification  No treatment | 1 (2.8%)  6 (16.7%)  29 (80.5%) |
| If you feed calves WM, to which age groups do you feed it?  0-2 weeks  2-4 weeks  >4 weeks  No age difference | 5 (13.9%)  3 (8.3%)  2 (5.6%)  26 (72.2%) |
| If you feed calves WM, to which groups do you feed it?  Males  Females  All | 8 (22.2%)  1 (2.8%)  27 (75.0%) |
| What feeding system do you use for calves?  Bucket  Automatic feeder | 33 (91.7%)  3 (8.3%) |
| How often do you clean the feeding equipment?  After each use  Once a day  Less than once a day | 34 (94.4%)  1 (2.8%)  1 (2.8%) |
| Are sick calves separated from healthy calves?  No  Yes | 12 (33.3%)  24 (66.7%) |
| Who mainly diagnoses calf diseases?  Veterinarian  Calf caretaker  Manager  Other | 9 (25.0%)  16 (44.4%)  9 (25%)  2 (5.6%) |
| Who mainly administers the antimicrobials to calves?  Veterinarian  Calf raiser  Manager  Other | 4 (11.1%)  20 (55.6%)  9 (25.0%)  3 (8.3%) |

**Supplementary Table 3.** Management of antimicrobial drugs

| **Question** | **Answer** |
| --- | --- |
| Does the farm record antimicrobial purchases?  No  Yes | 7 (19.4%)  29 (80.6%) |
| Does the farm record antimicrobial treatments in calves?  No  Yes | 7 (19.4%)  29 (80.6%) |
| Do you have protocols for antimicrobial use in calves?  No  Yes | 15 (41.7%)  21 (58.3%) |
| What antimicrobials do you use to treat resp infections in calves?*  Tetracyclines  Fluoroquinolones  Florfenicol  Macrolides  Penicillin/Streptomycine  Betalactam  Aminoglycoside | 19 (52.8%)  14 (38.9%)  8 (22.2%)  6 (16.7%)  2 (5.6%)  1 (2.8%)  1 (2.8%) |
| What antimicrobials do you use to treat diarrhea?*  Sulfonamide  Fluoroquinolones  Tetracyclines  Macrolides | 25 (69.4%)  14 (38.9%)  8 (22.2%)  1(2.8%) |
| What antimicrobials do you use to treat clinical mastitis?*  Cephalosporin  Betalactam  Tetracycline  Aminoglycoside  Fluoroquinolone  Sulfa  Macrolydes  Lincosamide | 23 (63.9%)  16 (44.4%)  7 (19.4%)  4 (11.1%)  1 (2.8%)  2 (5.6%)  3 (8.3%)  2 (5.6%) |
| What antimicrobials do you use for dry cow therapy?*  Betalactam x aminoglycoside  Cephalosporin  Betalactam  Rifamicin | 14 (38.9%)  10 (27.8%)  10 (27.8%)  2 (5.6%) |

*More than one group of antimicrobials can be used on each farm.
